# Valorization of Fruit and Vegetables Industry By-Streams for 3D Printing—A Review

**DOI:** 10.3390/foods13142186

**Published:** 2024-07-11

**Authors:** Alona Tyupova, Joanna Harasym

**Affiliations:** 1 Department of Biotechnology and Food Analysis, Wroclaw University of Economics and Business, Komandorska 118/120, 53-345 Wroclaw, Poland; tyupovaa@gmail.com; 2 Adaptive Food Systems Accelerator-Science Centre, Wroclaw University of Economics and Business, 53-345 Wroclaw, Poland

**Keywords:** food waste, food by-streams, 3D printing, vegetable ink, fruit ink, valorization

## Abstract

An energy supply crisis is impacting all the branches, including the agriculture and food industry. The wise and responsible utilization of plant raw materials already cultivated is becoming a must in the country’s economy. Not only the waste of the resources included but also the environmental challenge are concerns behind the not exploited food production by-streams and leftovers’ valorization. Fruits and vegetables’ out of the market quality “beauty” standards are still valuable sources of nutritious compounds. The conversion of raw materials into edible products can be provided by many techniques, with three-dimensional printing being the most individualized one. The main objective of this review was to summarize the existing efforts for the valorization of fruits and vegetable residuals into edible 3D inks and then 3D printed products. The clustering analysis was used for the separation of certain research approaches in fruit and vegetable wastes exploitation for 3D printing inks’ formulation. As the multilayer deposit technique is strongly dependent on the printing conditions and 3D ink formulation, therefore the tabularized description was included presenting the nozzle diameter, printing speed and other conditions specified.

## 1. Introduction

**Figure 1:**
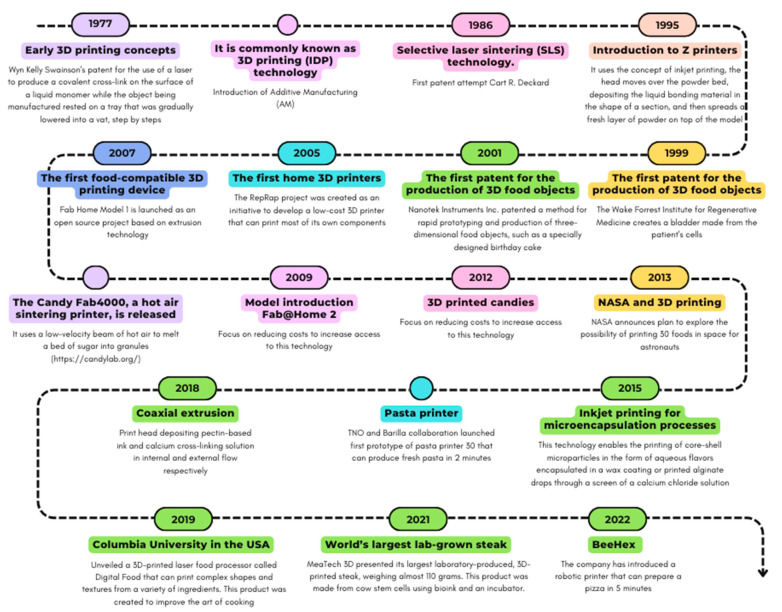
Chronological breakthroughs in the development of 3D food printing technology.

Three-dimensional (3D) printing is an emerging technology that has huge applications in the production of physical prototypes and the practical evaluation of product designs, health products and living biological systems. This is a manufacturing process that creates complex, solid (or semi-solid) shapes in a layer-by-layer process. Chemical reactions, phase transitions and other material properties are used to join the layers together [1]. Biotechnology, pharmaceuticals and medicine are just some of the possible applications of this technology; however, it continues to grow in other complex areas, such as food design and development [2]. The development of the food printing concept is shown in Figure 1.

The challenges facing the food industry in the coming years include the need to feed a growing global population, the increasing demand for personalized and healthy food, and the need to produce food in an affordable and environmentally friendly way [3]. In addition, according to the United Nations, 13% of food is lost between harvest and sale [4]. Radical innovations are needed to solve these problems, which creates a space for the enhanced implementation of digitally supported food production such as 3D printing. The emphasis on personalized nutrition options has led to a growing market that demands greater flexibility and responsiveness, qualities that cannot be easily achieved by traditional mass production methods.

New 3D printing technology (3DFP) in food is seen as a potential solution to meet these needs [5,6,7,8].

Food 3D printing has great potential to create personalized foods with specific properties such as nutrients, shape, texture, color and taste. Several 3D food printing technologies currently exist, but most research has focused on improving the quality of printed food rather than developing large-scale production technology. Further research is needed to develop effective and economical methods for 3D food printing on a mass scale. However, this particular technique has the potential to revolutionize the food industry by enabling the creation of personalized and high-quality products [9].

The use of raw material in 3D food printing is one way to combat the problem of proper food resources exploitation, which is becoming increasingly important worldwide. According to the UN, about 1.3 billion tons of food are wasted or lost every year, which is about one-third of all food produced. This not only harms the environment but also increases the risk of hunger and malnutrition for millions of people [10].

Some start-ups and researchers are developing 3D food printing solutions that use food waste to print food products, reducing food waste. Upprinting Food (2018–2021) is a Dutch startup that uses 3D printing to turn food waste into edible and tasty dishes. They collect waste such as bread, fruits and vegetables from restaurants and stores and process them into purees, which are then printed into various shapes and dried in an oven. The resulting products can be stored for several weeks and reheated before use [11]. These are just some examples of the use of waste in 3D food printing. The technology has great potential to reduce food waste, increase the nutritional value of food, enhance the taste experience and create new culinary possibilities.

However, there are some challenges and limitations, such as production costs, the availability of suitable materials and the need to meet strict sanitary standards. Future trends in 3D printing include the development of numerical simulations, the introduction of cooking-to-print technology and 4D modifications. Further research and technology development are needed to fully realize the potential of 3D printing in the food industry [12].

The purpose of this study was to analyze existing technologies for 3D food printing (3DFP) from the group of food waste (including by-streams and food leftovers), with a focus on improving the quality of printed food products. The study highlighted the need for scalable and cost-effective technologies for the production of 3DFP from food waste and discussed the challenges and potential solutions for the implementation of 3DFP on an industrial scale. The purpose of this study was to evaluate the impact of 3DFP on global food waste. This included conducting a statistical analysis of food waste, exploring the potential of 3DFP to reduce food waste through the innovative use of food by-products and surplus materials.

Also, the purpose of this study was to highlight the need for continued research and development in the field of 3DFP from food waste. The study highlighted the importance of ongoing research and development to address existing limitations and discover new potential applications, calling for increased investment in 3DFP research to drive innovation and technological progress.

## 2. Materials and Methods

To outline the research area of 3D food printing, a bibliometric analysis was carried out based on a literature review, which is a tool for displaying information by grouping the keywords used in scientific documents into clusters. This enabled a more detailed study of the topic and highlighted its main points of contact with 3D printing of food products from food waste. To apply a thematic approach to the bibliometric literature analysis, an extensive search of information in the Scopus database was conducted. A primary search was conducted to find articles on the use of 3D printing in food production that covered a wide range of sources.

Using a bibliometric mapping method, publications from 2018 to 2024 were analyzed and 606 articles on 3D printing of food products were found. Moreover, the search showed a more active development of research in 2022–2023 (329 articles), and 46 articles have already been registered since the beginning of 2024. From the information presented above, it is clear that 3D printing of food products is a popular and actively developing field of science. Bibliometric studies of articles were conducted using VOS Viewer 1.6.20 (CWTS, Leiden, The Netherlands) [13]. Starting with 606 documents, using the search criteria of “food waste”, “vegetable waste” and “fruit waste” resulted in 43 documents analyzed. Considering all those factors, we finally used 22 research articles, 8 review articles, 8 conference papers, 1 book, 1 book chapter and 1 short survey.

According to the diagrams in Supplementary Figure S1A,B, we can see the connection between 3D printing and food waste, but looking at these connections, we can only conclude that by using 3D printing, it is possible to reduce the amount of waste created during food production, and this production is economically viable.

Since the first diagram shown in Supplementary Figure S1A,B is too broad and difficult to follow the main topic of the study, it was decided to create a diagram that focuses on articles on 3D printing of food products from vegetable and fruit waste.

**Figure 2:**
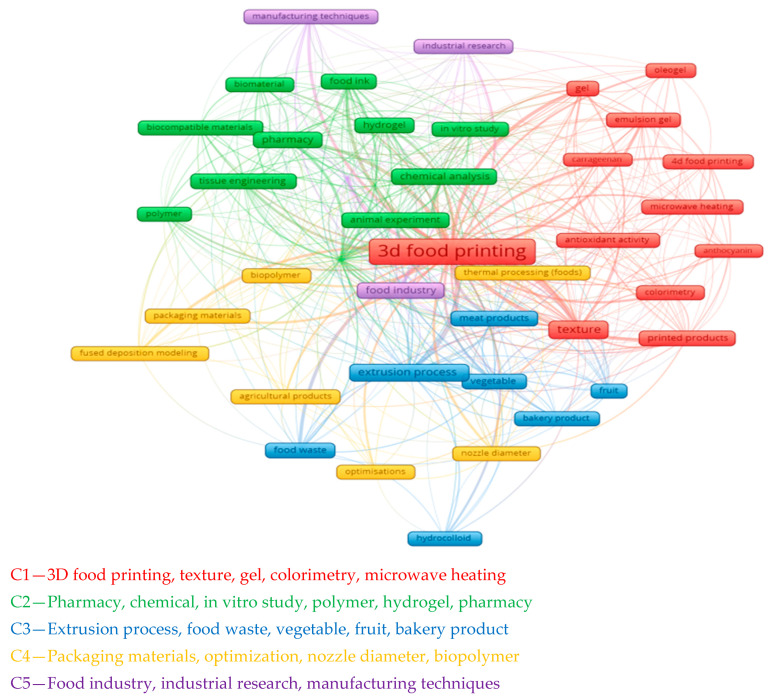
Clustering analysis of articles on 3D printing from waste.

Figure 2 shows a connection diagram related to 3D printing of food products. It is an interesting and promising technology that can have many applications in various industries, such as healthcare, the food industry, agriculture and others (C1,C2). The diagram shows various aspects and categories related to 3D food printing, such as the following:

- Food products that can be printed with 3D printers, such as desserts (cookies, chocolates of various shapes), pizza, etc. (C1);
- Manufacturing processes used in 3D food printing, such as extrusion, inkjet printing, microwave heating and others (C1, C2, C3, C4);
- Materials used as carcasses for 3D printing of food, e.g., vegetables, fruits, processed meat and fish, bakery products, food waste, hydrogels and others (C1, C3);
- Analyses conducted to evaluate the quality and properties of 3D printed food products, e.g., organoleptic, rheological, microbiological, colorimetric and others (C1, C2);
- Through the diagram, it can be seen that research in food 3D printing innovations is actively ongoing (C5).

Lines of different colors and thicknesses connect these terms, showing the strength or frequency of their connections. The central term is “3D food printing,” highlighted in red and in large font, indicating its importance.

Food waste can be reduced or reused through 3D food printing (C1, C4), which allows new shapes, structures and textures to be created from different food materials. The fused deposition modeling (FDM) method can be used to produce food products of different brands when using food bioink based on chocolate, cheese, honey and other products with the right thermal and rheological properties.

Agricultural products such as fruits, vegetables, grains, beans, etc., are sources of organic substances such as cellulose, starch, sugars, proteins, fats, vitamins and minerals. These substances can be converted into other useful products such as biogas, compost, ethanol, methanol, glycerol, lactate and others through various biochemical processes such as fermentation, hydrolysis, oxidation and esterification. These processes use biocatalysts such as enzymes, microorganisms, cells and tissues to speed up chemical reactions.

It is clear that food waste is related not only to food itself but also to packaging materials and processing. It can be said that this cluster (C3, C4) has the potential to recycle and reuse resources through biochemical processing, as shown in Figure 2.

The “fruit” cluster is related to terms such as “texture”, “3D food printing”, “printed products” and “colorimetry” (C1,C2,C4). This implies developments in the study of fruit texture, color and food printing methods using fruit ingredients. In a chemical context, this may involve analyzing the chemotype of fruit products, their nutritional value, and ways to optimize recipes for preparing inks for 3D printing (C1). Printed products can be used for various purposes, such as personalized nutrition, medical nutrition, alternative protein sources, waste reduction and others. Various chemical methods such as spectroscopy, chromatography, titration, potentiometry and others can be used to evaluate the quality and properties of printed products. The texture of fruit inks can be determined by rheological properties such as viscosity, elasticity, plasticity, hardness, adhesion and cohesion. These properties depend on the composition, structure and interaction between polysaccharides, proteins, lipids and water.

**Figure 3:**
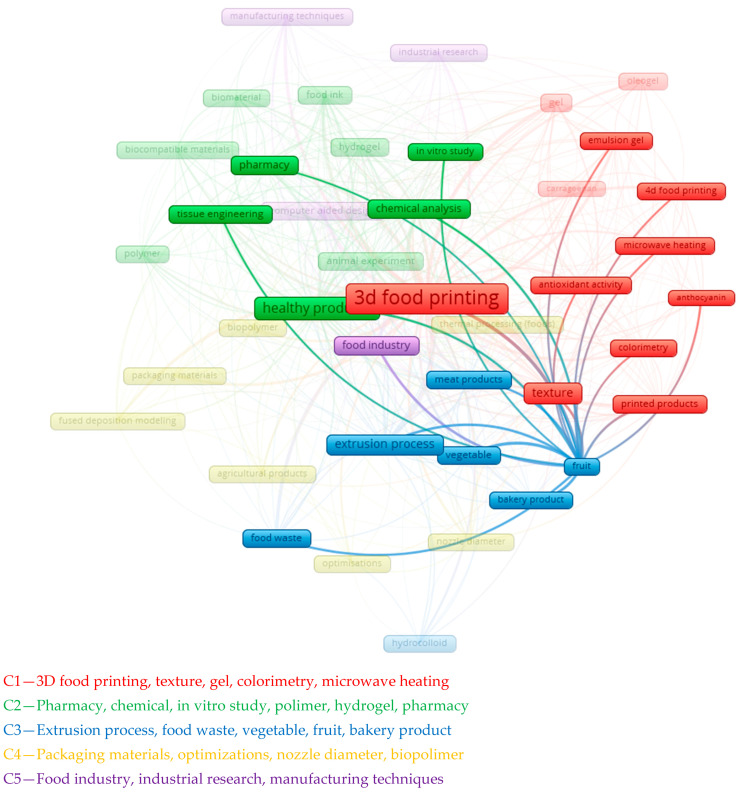
Clustering analysis of articles on 3D printing from fruit waste.

The texture of fruit inks affects the structure of the final product, as well as consumer perception, influencing taste, aroma and satiety. Three-dimensional food printing is a cutting-edge technology that makes it possible to create intricate structures from various food materials. For this purpose, food bioinks are needed, which include ingredients such as fruit purees, juices, pastes, gels, emulsions, powders and others, as shown in Figure 3.

The “vegetables” cluster is associated with concepts such as “texture”, the “extrusion process”, “food waste”, “3d food printing” and “bakery products” (C1, C3). In order for vegetables to participate in the 3D food printing process, they need to be given a certain texture (C1). This depends on their composition, texture and moisture content.

**Figure 4:**
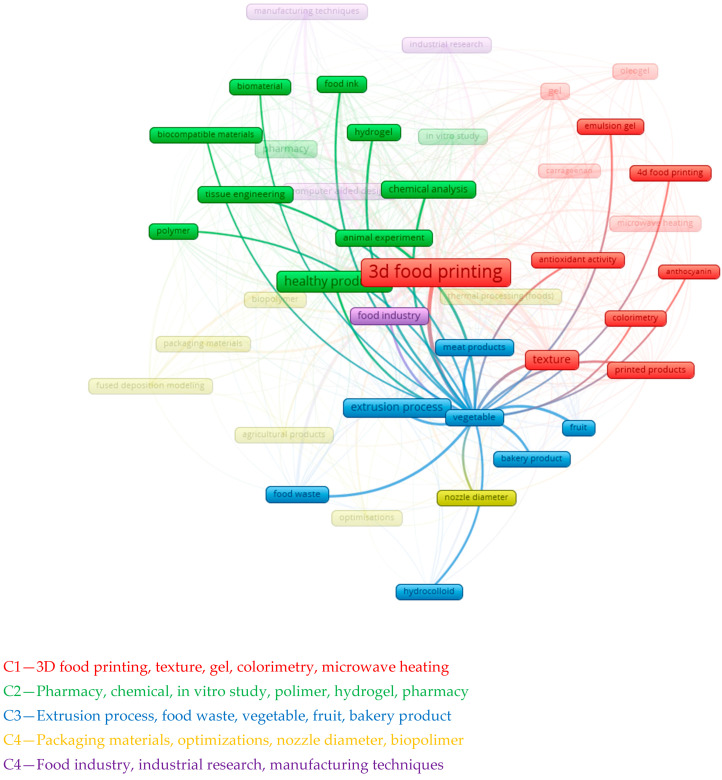
Clustering analysis of articles on 3D printing from vegetable waste.

Vegetables contain various organic substances, such as cellulose, starch, pectin, proteins, fats, vitamins and minerals, which affect their firmness, chewiness, elasticity and crispness. The texture of vegetables can change during handling, storage and cooking and is influenced by their temperature, humidity and pH, as shown in Figure 4.

## 3. Microbial Safety Aspects of Plant-Based 3D Printed Food

While various technologies have been explored for 3D food printing, including jet binding and laser sintering, extrusion technologies are by far the most widely used. Extrusion systems for depositing food materials can utilize different configurations such as cartesian, polar, delta and SCARA. These configurations describe the movement of the printer’s head and/or bed within the X-Y-Z space, enabling the precise deposition of food materials according to CAD models [14]. The extrusion process is one 3D printing method that uses a nozzle to extrude food bioink in the form of a thread or jet. This method can be used to produce food products from plant raw materials, such as purees, juices, pastes, gels and others. For this purpose, it is necessary to take into account the rheological properties of plant bioinks, such as viscosity, plasticity, elasticity and thixotropy, which depend on their composition, concentration, temperature and strain rate. Also to be considered is the nozzle diameter, which affects the shape, size and quality of printed products [15,16,17].

Food waste is an environmental problem because it causes pollution and greenhouse gas emissions and wastes resources. Vegetables and fruits are among the most wasted food items due to their short shelf life, high moisture content and low microbiological resistance. Therefore, the study of the use of vegetable and fruit waste, as well as its unfitness for use, is a topic of great importance, although underdeveloped.

Microbial safety when converting the food residuals into bioink raw materials is another issue. Addressing these concerns is vital to prevent contamination, spoilage and pathogen-related health risks, thereby ensuring the production of safe and high-quality 3D printed food products. Ensuring the microbiological safety of the food residuals and by-streams used as raw materials for 3D food printing involves a multi-faceted approach that includes stringent hygiene practices, effective processing and storage methods, regular microbiological testing and compliance with regulatory standards. The search performed on the SCOPUS databank with the keywords [3D Food Printing AND microbiol*] retrieved only 29 documents, of which only 3 address the microbial safety of 3D printed food. Severini et al., 2018, who printed raw materials such as carrots, pears, kiwi fruit, broccoli raab leaves and avocado which were purchased locally and stored in refrigerated conditions at 4 °C until the experiments for a maximum of 2 days, observed that a concentration in bacteria of 4.28 Log CFU/g was noted after printing [18]. Markovinovic et al., 2023, aim to develop a strawberry-based functional product by incorporating two hydrocolloids (corn and wheat starch) at three different concentrations (10%, 15% and 20%). It will investigate the impact of 3D printing process parameters on the product’s physicochemical and textural properties, bioactive and antioxidant potential, and microbiological stability, both with and without the addition of natural antimicrobial agents. Microbial analysis was performed during the 10 days of storage at 4 °C. The samples with added vanillin (1 g/L) remained stable during the 4-day storage period, but an increase in bacterial counts above the desired level was observed on days 7 and 10. Interestingly, in samples with a higher vanillin concentration (1 g/L), a high bacterial count was only observed on the 4th day, after which the count remained within the desired range. These results suggest the possibility of the inadequate homogenization of the microbial agent within the strawberry matrix, leading to localized concentrations of the inhibitory agent and areas with reduced antimicrobial activity. Additionally, a reduction in aerobic mesophilic bacteria was observed at the citral concentrations tested during storage, indicating citral’s potential inhibitory effect on bacterial growth. The presence of aerobic mesophilic bacteria before storage likely resulted from handling during product preparation and was limited to the surface. The inadequate homogenization of the antimicrobial agent could lead to varying citral concentrations within the sample. Future studies should address the technical challenges of delivering antimicrobial agents in such products. Our results indicate that the product formation process is free from pathogenic contamination and that adding citral (75 mg/L) provided the best microbiological quality. However, manufacturing methods need to be refined to ensure uniform antimicrobial activity throughout the final products [19].

Abedini et al., 2024, verified the microbial safety of a plant-based 3D printed hamburger. Texturized soy protein was soaked in water for 4 h, then drained and ground using a meat grinder. Ground soy protein was mixed with gum, onion, gluten, bread flour, oil, spices and salt. The ingredients were thoroughly blended. This study utilized two sample types (printed and conventional) for a comprehensive evaluation. The safety indicators were assessed under two storage conditions: refrigeration and room temperature. The refrigerated samples were tested on days 0, 5, 7, 12 and 14. The samples stored at room temperature (20 to 25 °C) were tested at 0, 24 and 96 h after printing. Examination of the refrigerated samples revealed a significant increase in microbial count over the 14-day study period. By days 7, 12 and 14, both the 3D printed and conventional samples exceeded 5 log CFU/g. The conventional method samples had higher microorganism levels, suggesting cross-contamination during handling. One reason for the increased microbial growth in the conventional method is the greater use of hands compared to the 3D printing method. Although both the 3D printer and conventional sanitation devices are effective, the difference in machine materials may significantly impact contamination transfer. PTC (psychrophilic total count) analysis showed no significant difference between the 3D printed and conventional samples on day 1, with levels not detected. However, by day 7, the PTC levels had increased significantly in 3D printed samples. Additionally, on days 7 and 12, the PTC levels in 3D printed samples were significantly higher than in conventional samples, indicating that psychrophilic bacteria were the dominant microorganisms in the 3D printed samples. Examination of the samples stored at room temperature showed a significant increase in the TVC (total viable count) in all samples over the 96 h storage period [20].

Even when using fresh fruits and vegetables, rather than by-streams or leftovers, it is crucial to ensure the sanitization of all components that come into contact with the food before applying 3D food printing in restaurants and at an industrial scale. In addition to technical challenges, it is important to recognize the critical need for detailed experiments focused on the safety risks associated with 3D printed food. Surprisingly, these experiments have been largely overlooked, despite the fact that 3D printed food and 4D food are considered ‘novel foods’ under the Novel Food Regulation 2283/2015. This regulation states that novel foods must be safe for consumers, properly labeled to avoid misleading information and not nutritionally disadvantageous if they are intended to replace existing foods [21]. While common processing methods (both thermal and non-thermal) may ensure the microbiological and nutritional quality of food formulas before printing, the involvement of food industries capable of producing safe, stable and printable food formulas is crucial for the successful market implementation of 3D food printing.

## 4. Fruit- and Vegetable-Based 3D Printing Ink

In the field of 3D food printing, the materials used for printing are usually called “Food Inks”. These are specially prepared ingredients that can be used to print various food shapes, textures and colors [22,23]. Edible inks can be made from a wide range of products and ingredients, such as milk, cheese, oils, eggs, chocolate and many others. They are mixed in specific proportions and added to special cartridges, which are then used in 3D printing equipment. Before use, the materials are treated to ensure product safety and hygiene [7,23].

However, disadvantages such as lack of fluidity and material properties unsuitable for 3D printing processes make most food materials unsuitable for 3D printing. At the same time, non-food polymer-based materials are capable of reproducing almost any art form specified by researchers. The main advantage of these materials is their high stability and predictable processing behavior. In the food industry, there are few materials with similar properties due to their “safe use limits” and “allowable limits” in food products [24].

Food materials for 3D printing are divided into the following:

- For printing: hummus and chocolate. These materials do not require additional processing before printing [25,26];
- Non-printable materials, such as rice, fruits and vegetables, and meat, require pre-processing to be used effectively in 3D printing [27].

The printed material must be designed to flow smoothly through the nozzle (nozzle tip) and provide a strong structure to the final product [28]. Vegetables and fruits are considered non-printable foods due to their high water content and lower carbohydrate, fat and protein content [29]. Therefore, it is necessary to monitor parameters such as yield strength, the consistency index and the elastic modulus in order to improve extrusion and extend the time for product shape retention [30]. In addition, the addition of polysaccharides and other additives to inks of different compositions will predict the suitability of the ink with up to 89% accuracy [31,32].

Waste from the fruit and vegetable industry consists not only of pollutants but also of organic materials such as fruit peels, seeds, juices, etc., and 30–50% of these by-products are further used to produce biogas, compost and fertilizers. Various food additives are also successfully extracted from the waste [33]. However, in our research, we will explore the use of vegetable and fruit waste in 3D food printing.

### 4.1. Fruit-Based 3D Printing Ink

Fruit waste such as mango peels, pomegranate peels, orange peels, apple seeds and pineapple peels can be used for food 3D printing. These fruit wastes contain bioactive substances that can be extracted and used to improve the nutritional and flavor profiles of various food products, such as baked goods, pastries, yogurts and cookies (Table 1). Using innovative technologies, including extrusion, these fruit wastes can be transformed into functional ingredients that produce new food products with improved properties. Various studies have also demonstrated the variety of the bioactive ingredients contained in fruit waste and their potential to improve human health [34].

Research has shown that lemon jelly with potato starch is suitable for 3D printing. Studies on the quality of lemon juice gel have shown that the concentration of starch plays a key role in the structure and properties of the gel. Optimizing printing parameters, such as nozzle size and extrusion speed, can improve the quality of 3D designs [35]. The results of a banana paste supplemented with pea protein isolate showed that it improves printing performance and reduces the formation of “tails” that interfere with continuous extrusion. Also, the use of high starch content (17.5, 20 g/100 g) led to more consistent properties. An optimally balanced pea protein isolate content in the paste is important for achieving the desired fluidity and adhesion in 3D printing [32].

A study has shown that 3D printing can turn orange peels into an edible snack. Biochemical analysis confirmed the preservation of the antioxidant properties of orange peel waste. The study showed that the ink produced from the waste is safe to use. Overall, the study highlights the potential of using 3D printing to create food products from food waste, promoting sustainability in food production and consumption [36,37].

The addition of orange products to 3D printed gels was also investigated. The additive was found to improve the flexibility and printability of the gels and increase the fiber content of the snacks, helping to reduce food waste and promote healthy eating. However, some samples failed due to increased stiffness and printing difficulties. Overall, the use of orange products not only improves the ability to print gels but also allows the creation of valuable food products from food industry waste [37].

A study was conducted to show that the addition of orange by-products affects the color and quality of gels. Various parameters of the gels were analyzed, including their rheology, bioactive content, cutting force and color characteristics. The study confirmed the promise of using orange by-products to improve the flexibility and printability of gels, as well as to create valuable food products from industrial waste.

Some problems were also identified related to the stiffness and difficulty of printing some samples and changes in their color characteristics. Further research is needed in the future to improve the printing process and expand the range of food products that can be made using orange by-products [38].

**Table 1:** Fruit-based inks and 3D printing using fruits residuals.

Raw Materials	Processing Conditions	Ink Type	3D Printing Conditions	Printed Product	Ref.
Banana paste	Grind dried bananas using a high speed blender. Addition of pea protein isolate to improve 3D printing performance and reduce tailing effect when extruding banana paste. Banana paste and PPI are sifted through a 300 micron sieve. L-ascorbic acid is added to the resulting mixture as an antioxidant. Everything was thoroughly mixed until the powders were uniformly combined. The powder is dissolved in distilled water in a 1:1 ratio; the mixing process was carried out manually for 10 min. The resulting pastes were stored at 4 °C overnight and used for experiments the next day.	paste type ink	Layer height 1.1 mm The height of the first layer is 0.9 mm Nozzle speed 40 mm/s Temperature 25 ± 1 °C Nine perimeters of vertical shells after preliminary experiments.	Two wedge-shaped pieces facing each other and forming a 30° angle of inclination	[32]
Orange peel waste	Orange peels are dried in an oven at 60 °C for 24 h. The dried peel is ground into powder using a kitchen blender at 28,000 rpm for 10 min. The resulting powder is passed through a chopping sieve with a diameter of 300 mm. To prepare the ink, the prepared powder is added to distilled water with the addition of xanthan gum in various concentrations from 0.4% to 1.0%. All paints are thoroughly mixed using a planetary centrifugal mixer for 5 min at 2000 rpm and a temperature of 25 °C.	direct ink writing (DIW)	The printing process was carried out at room temperature, in a sterile environment, using a 50 mL dispensing syringe with a 20 G nozzle calibrated to a layer thickness of 0.40 mm. All samples were loaded onto glass substrates before printing, taking into account printing speed and ink pressure. Pneumatic Printer: Direct Ink Printer (DIW). Software: MuCAD V (controls speed and print path of DIW printer); Program: Solidworks (creating 3D models and exporting to STL format); Software: Slic3r (splitting the model into layers and creating G-code for 3D printers). Calibrate the distance between the substrate and the nozzle using a measuring tool. Control the printing speed and extrusion pressure during the printing process. Print at room temperature in a sterile environment.	Food topping Logo display Food bowl Mesh cube	[36]
Orange peel waste	The OPW was then dried in an oven heated at 60 °C for 24 h. Dried OPW was ground into OPW powder using a 2000 W kitchen blender at 28,000 rpm for 10 min. Next, the OPW powder was sifted into fine particle size (Industrial and Laboratory Consumables, China). OPW ink samples were prepared by adding OPW powder into deionized water at different formulations.	DIW	A pneumatic-extrusion-based DIW printer (SHOTmini 200 Sx, Musashi Engineering, Inc., Tokyo, Japan) in a chamber to preserve a sterile environment. MuCAD V software (Musashi Engineering, Inc., Tokyo, Japan). Solidworks (Dassault Systèmes, Waltham, MA, USA), a computer-aided design (CAD) software, to create a 3D model. Slic3r, open-source software to create G-code for 3D printers. 50 mL Luer lock dispensing syringe with a 20 G nozzle (Birmingham Gauge) (V–S liquid control equipment, China). The layer thickness was 0.40 mm and the printing speed and dispensing pressure were 50 mm/s and 0.090 MPa, respectively. All of the printing was conducted at ambient temperature.	SUTD LOGO, food toppings, soup bowl, biscuit, crackers	[37]
Apricot gel with added orange zest	To prepare the inks for 3D printing, the study employed a Moore 2 Pro Clay 3D printer from Shenzhen Tronxy Technology Co., Ltd., Shenzhen, China, utilizing Fused Deposition Modeling (FDM) extruder technology. Designed a 3 cm diameter and 1 cm height cylinder using Tinkercad software and set the printing parameters using the Ultimaker Cura software v.5.1.1.	-	The printing process used Fused Deposition Modeling (FDM) extrusion technology, a precise X-Y-Z positioning system and a stepper motor-driven extrusion system. The printing was carried out with a rectilinear infill of 100%, a layer height of 1.2 mm and a speed of 20 mm/s, using a 1.2 mm diameter nozzle. The rheological behavior of the printed inks was evaluated both before and after the 3D printing process to assess changes and provide insights into the structure and stability of the printed food. After printing, the samples were frozen for 24 h at −45 °C and then dried in a lyophilizer for 48 h at −56.6 °C to obtain the final products.	Control sample	[38]
Grape pomace	Mix: powdered sugar 50 g shortening (45 g) vanilla essence 0.3 g A mixture of refined wheat flour and grape pomace powder in ratios 100:0, 98:2, 96:4, 94:6, 92:8 by weight	-	Nozzle diameter 1.28 mm Extruder motor speed 600 rpm Print speed 400 mm/min Extrusion food 3D printer CARK Post-process at 130 °C for 12 min	Value-added functional cookies	[39]
Strawberry tree fruit	Washed and stored at −18 °C. Before the experiment, defrosted to +4 °C. Grind with a home homogenizer to obtain a homogeneous mixture with a uniform particle size (d ≤ 4 mm). Starch carriers, mainly wheat starch and corn starch, were added in amounts of 4%, 6% and 8% (by weight), respectively.		3D printer: Foodini^®^ (Natural Machines, Barcelona, Spain) Nozzle diameter: 4 mm Program 1: printing speed—8000 mm/min; printed line thickness—3.5 mm mixture consumption—1.4; height of the nozzle of the first layer—6 mm Program 2: printing speed—14,000 mm/min; printed line thickness—3.4 mm mixture consumption—1.65; the height of the nozzle of the first layer is 4.5 mm	Heart shape made of three layers	[40]
Durian husk	Durian husk ink with a particle diameter of 100–300 μm (25% *w*/*w*) required the addition of 5% *w*/*w* xanthan gum to achieve extrusion from the nozzle. In contrast, reducing the diameter of durian husk particles (<100 μm, 25% *w*/*w*) enabled printing with additive-free inks.		A pneumatic-extrusion-based DIW printer (SHOTmini 200 Sx, Musashi Engineering, Inc., Tokyo, Japan) in a chamber to preserve a sterile environment. MuCAD V software (Musashi Engineering, Inc., Tokyo, Japan). Solidworks (Dassault Systèmes, Waltham, MA, USA), a computer-aided design (CAD) software, to create a 3D model. Slic3r, open-source software to create G-code for 3D printers. 50 mL Luer lock dispensing syringe and fitted with a 16G and/or 20 G nozzle (Birmingham Gauge) (V–S liquid control equipment, China). The printing speed was 20 mm/s. Dispensing pressure was adjusted using a pressure dispenser (ML-5000XII, Musashi Engineering Inc., Tokyo, Japan). All of the printing was conducted at room temperature.	20 × 20 mm square	[41]
Lemon juice gel	Lemon juice is mixed with different contents of potato starch (10, 12.5, 15, 17.5, 20 g per 100 g). The mixture was completely mixed with a mixer (ULTRA-TURRAX^®^ IKA^®^ T18 basic, Model: T18BS25, Germany) and steamed for 20 min at a temperature of 86 ± 2 °C. During cooking, the container was covered with food-grade film to prevent water loss. The resulting sample was then cooled to room temperature and stored at 4 °C to form a weak jelly-like structure.	DIW	Nozzle diameter 1 mm Extrusion speed 24 mm^3^/s Nozzle speed 30 mm/s	Control sample	[35]

In addition, research has been conducted on using food industry waste in the 3D printing process to create cookies. This method makes it possible not only to produce products with individual shapes but also to overcome possible consumer aversion to products made from waste. Organoleptic tests have been conducted to assess the potential of printed cookies. These studies allow us not only to assess the quality of printed products but also their organoleptic characteristics, which is important for understanding consumer preferences and improving production processes [39].

According to the study, the fruits of *Arbutus unedo* L., or the fruit of the strawberry tree, contain high levels of bioactive compounds that can act as antimicrobial agents that can protect the integrity of DNA. Using these fruits to produce functional foods using 3D printing technology could lead to innovative functional products [40].

From the study, it can be concluded that optimizing printing parameters such as nozzle size, extrusion speed and material properties is key to improving the quality of 3D designs. The importance of material composition and the rheological properties in the 3D printing process was emphasized, indicating the need for materials with adequate flow properties and structural support.

### 4.2. Vegetables Waste-Based 3D Printing Ink

Vegetables are being used in incremental manufacturing, such as 3D food printing, to increase their value and optimize the use of food resources. As a result of strict industry standards and a short shelf life, vegetables and their parts, such as stems, peels and vegetable scraps, are often discarded in the trash. Instead of discarding this waste, it can be transformed into products with significant added value, such as edible ink, which are then used to create beautiful and nutritionally optimized dishes, especially for specific categories, such as patients with dysphagia. Additive manufacturing technologies, such as 3D food printing, are helping to increase the waste-free use of vegetables, improve food safety and help create sustainable solutions to reduce food waste (Table 2).

The suitability of food inks for printing was evaluated based on the ability to retain structure for 30 min and the syneresis of food inks. Printing parameters, such as printing speed and extrusion speed, were optimized and determined before printing. The design of the hexagonal prism was previously stored in the FOODINI database [42].

Experiments to use automated 3D printing to create food products have yielded interesting results. It turned out that adding broccoli and carrot powders instead of wheat flour in different proportions causes changes in the properties of printing inks and affects the accuracy and quality of printing. One of the key success factors in 3D printing is maintaining ink consistency and eliminating air pockets in the cartridge. It is important to adhere to certain printing settings that have been established through initial experiments with different carrot and broccoli compositions. Geometric characteristics of the printed objects, including volume, were used in the process of evaluating printing accuracy. Their analysis showed that the volumes of the 3D printed samples varied, and some of them differed from the volumes of the 3D digital geometry. The deviations in volume indicated a lower manufacturing quality [43].

However, the researchers were able to achieve good shape retention using 75% carrots and broccoli. It should be noted that the structural stability of the broccoli samples left much to be desired, leading to some volume deviations compared to the digital 3D geometry. This underscores the importance of die strength for successful 3D printing. Combining carrots and broccoli with wheat flour significantly improved dimensional stability. The best results matching the digital file with minimal geometric errors came from using 50% and 75% vegetable composition. However, with less vegetable content (25%), the paste structure did not allow a good shape after printing [43].

**Table 2:** Vegetable-based inks and 3D printing using vegetable residuals.

Raw Materials	Processing Conditions	Ink Type	3D Printing Conditions	Printed Product	Ref.
The stems and stalks of kale and spinach	Refrigerate at 4 °C for a maximum of two days before use. Washed and crushed by hand. Cook until tender for ~15 min (spinach) and ~45 min (cabbage). Squeeze out and drain the remaining water. The boiled stems and stalks were ground separately in a food processor for 5 min to obtain a puree-like consistency. Nine edible inks have been created.	DIW	Food 3D printer based on extrusion FOODINI (Natural Machines, Spain). A nozzle size of 1.5 mm was used to print a 9 mm high hexagonal prism in six layers.	a 1 × 1 cm red square	[42]
Specifically broccoli and carrots	Stored in the refrigerator for no more than 2 days cut into small pieces (~4 cm in size). Blanched with steam in a steamer (Dixie, M-6 steam blancher-cooler) at 90 °C for 3 min to inactivate peroxidase. Packaged in Ziploc bags and frozen for at least 24 h. Freeze-dried at −45 °C and 7.3 Pa (LABCONCO) for 48 h. The dried products were ground into a fine powder using a Blizer 2 food processor (Robot Coupe USA Inc.). Sifted on a Meinzer II sieve shaker with 250 µm holes for approximately 15 min. Stored in the refrigerator (4 °C) until further use.	DIW	Extrusion food 3D printer (Foodini, Natural Machines, Spain) Nozzle diameter 1.5 mm four layers 6 mm high	Snack cracker	[43]
Pumpkin flour/ purple potato powder/spinach powder/sorghum flour/carrots	All samples were stirred at room temperature for 30 min using a magnetic stirrer homogenized for 5 min using a homogenizer (IKA^®^, T18 Basic Ultra Tur-rax^®^, Staffin, Germany) at 6400 rpm. Samples A and G were exposed to a 70 °C water bath (SSW-420–2S, Shanghai boxun Medical-biological Instrument Co., China) for 20 and 15 min, respectively.	DIW	Extrusion rate 20–22 mm/s^3^ Nozzle movement speed 15–20 mm/s Fill rate 80%	Cone and cylinder with a diameter of 30 mm	[44]
Spinach puree, tomato puree and apple sauce	The water content of tomato, apple and spinach purees was 88.6%, 88.8% and 92%, respectively. Homogenized using a hand blender. Placed on a magnetic stirrer for 6 h for complete homogenization.	DIW	The DIW printer uses compressed air (Ultimus V air pressure 105 controller, Nordson EFD, East Providence, RI, USA) to extrude the inks through a tapered 106 nozzle onto a build plate. Lines 175 mm in length of each ink were extruded through three different nozzle diameters: 16 gauge (1.194 mm), 18 gauge (0.838 mm) and 20 gauge (0.603 mm).	Octopus, pyramid and cubic	[45]
Potato peelings	Air-dry potato peels were obtained by grinding the dried samples in a Preethi Blue Leaf Platinum household mixer mill (750 W). Sieved using 0.125 mm and 0.325 mm sieves to separate into two different fractions. Guar gum. Whole wheat flour (77 g carbohydrates, 10.9 g protein, 1.7 g fat and 10.5 g dietary fiber; per 100 g), salt, vegetable oil.	DIW	Nozzle diameter (0.5, 0.82 and 1.28 mm); extrusion motor speed (180 and 240 rpm); printing speed (300, 600, 900, 1200 mm/min) Constant pressure 4 bar; the noodles were post-steamed at 100 °C and 1 atm pressure for 5 min, followed by drying in a laboratory hot air oven for 2.5 h at 68 °C to final moisture content. Below the limit.	noodles	[46]
Tomato paste	Tomato puree was centrifuged at 20 °C with a force of 1000–10,000× *g* for 20 min using a Sorvall Lynx 4000 (Thermo Scientific, Pullman, WA, USA) with a fixed angle rotor F14–14 × 50 s. After centrifugation, the precipitate was collected and used for 3D printing and further rheological analysis.	DIW	ByFlow 3D printer (byFlow BV, Netherlands). Print at ambient temperature. Slic3r Software; Extrusion nozzle: gray tips with a diameter of 1.2 mm and green tips with a diameter of 0.8 mm Nordson Smooth Flow, Nordson Corporation, USA.; With green nozzle, print speed 25 mm/s; With gray nozzle, print speed 18 mm/s. The height of the printed layer was set to 0.6 mm.	a hollow square column with a bottom size of 30 × 30 mm and a wall thickness of 2.4 mm	[47]
Pumpkin paste	Pumpkin flakes were mixed with the prescribed amount of filtered tap water by a rubber spatula. To prevent clogging during 3D food printer extrusion, the prepared mixture was backed through a sieve with a mesh size of 1.18 mm, and the backed pumpkin paste was used as food ink for the 3D food printer.	DIW	A screw-based 3D food printer FP-2500 (SEIKI Corporation, Yonezawa, Japan) syringe with 2 mm nozzle. G-code file was read using the control software, Pronterface version 1.6.0. The room temperature where the 3D printer was installed and the temperature of the paste were set to approximately 25 °C.	cubic	[48]
Carrot/squid	Ten types of ink with different compositions were prepared	DIW.	Shapes designed using FreeCAD and then sliced with Ultimaker Cura software (Utrecht, The Netherlands). Printed using a pneumatically driven extrusion-based bioprinter (Baobab Root-1; Baobab Healthcare Inc., South Korea) with a syringe capacity of 100 mL. The temperature of the syringe filled with food ink and printing bed was kept constant at 25 °C using a temperature controller system. The printability of each ink was investigated at two different printing speeds (300 and 500 mm/min) and air pressure between 3 and 50 kPa using five different nozzles (12, 14, 16, 18 and 20 G).	Four layers of lattice scaffold (20 mm × 20 mm, infill density = 50%), ten layers of cubes (20 mm × 20 mm, infill density = 80%), and ten layers of hollow cylinders (outer diameter; OD = 25 mm, inner diameter; ID = 20 mm, infill density = 80%)	[49]
Spinach stems/kale stalks	The leafy components of kale and spinach were removed to obtain the stems and stalks, which were then chilled at 4 °C for up to two days until use. The stems and stalks were rinsed, manually chopped and boiled until tender—approximately 15 min for spinach and 45 min for kale. Excess water was squeezed and drained from the boiled vegetable wastes. The boiled stems and stalks were then blended in a food processor for 5 min until a puree-like consistency was achieved. This process created nine food inks: five with spinach puree, three with kale puree and one with a mixture of both, designated as ink SK. The final food inks were sieved with a ≤2 mm sieve to prevent the printer nozzle from clogging.	DIW	An extrusion-based 3D food printer, FOODINI (Natural machines, Spain), was used. A nozzle size of 1.5 mm was utilized to print. Print settings—Nozzle size (mm) 1.5; Print speed (mm/min) 3500; Ingredient flow speed 1.7; First ingredient hold (mm) 4.2; First layer nozzle height (mm) 1.4; Ingredient hold (mm) 3; Line thickness (mm) 1.4; Distance between layers (mm) 1.4; Fill factor (%) 1 First layer speed (%) 100.	A hexagonal prism with a height of 9 mm with six layers	[50]

There is a direct relationship between zero viscosity, Young’s modulus and printing height when using edible inks for 3D printing. The storage of the modulus is correlated with the stability of the shape of the printed samples, which affects the printing efficiency. The material’s stiffness and resistance to deformation affect its ability to retain size and shape after printing. Young’s modulus is positively correlated with printing height, indicating that stiffer materials have a higher printing height. Low-frequency NMR helps observe the movement of water molecules in different inks, and the changing distribution of water affects the microstructure and performance of 3D printing [44].

Therefore, further research in automated food 3D printing should focus on optimizing material composition and printing parameters to achieve the best possible results in terms of accuracy, quality and shape retention. This opens up new prospects for innovative food technologies and the creation of customized, specialized products [42,43,44,45,46].

In the study, we measured the particle size distribution of tomato puree using laser diffraction analysis. The shape and structure of the material’s cell wall were also examined using a light microscope. To prepare the tomato concentrate for 3D printing, the tomato puree was centrifuged and the soluble sugars in the supernatant were measured. The dry matter content and density of the resulting paste were then determined three times. The relative volume fraction of solids in the supernatant after centrifugation was also calculated and rheological measurements were taken. The stability and dispensability of the 3D printed materials were then investigated by conducting 3D printing experiments and measuring the force required to extrude the materials. All these measurements and experiments were carried out according to specific protocols and procedures [47].

The density, dry matter content, relative volume proportion and Brix number of the supernatant of the respective tomato puree and centrifuged pellets were then measured. The pellet density and Brix number of the supernatant were found to be constant for the tomato puree and paste, regardless of the centrifugation force used. The dry matter content of the pellets increased slightly, while the relative volume proportion almost doubled with increasing centrifugation force. The resulting paste appeared denser compared to the original tomato puree. Rheological analysis showed that melt flow stress, maximum G′, maximum G″ and zero shear stress viscosity increased with increasing centrifugation force. It was found that the breaking stress of the printed tomato puree depended on the pretreatment and was proportional to the flow stress, elastic modulus and zero shear stress viscosity. The failure stress index was higher with a larger nozzle than with a smaller nozzle. It was also found that the rupture stress was proportional to the stress flux, the value of the storage modulus and the viscosity at zero shear stress for both nozzle sizes. However, only the value of the storage modulus changed systematically with the stability of the printed structure, while the value of the lost modulus remained stable. Thus, the rheological properties of tomato paste may affect its printing stability [47].

We described the viscosity of the material and its effect on the stability of printing. It can be observed that the dependence of the rheological parameters on the calculated stresses changes depending on the diameter of the nozzle. This cannot be explained by material properties. Possible reasons are the increased stiffness of the structure with a larger nozzle, increased risk of nozzle clogging, increased complexity of the printing process, etc. A linear relationship between the measured extrusion pressure and measured melt stress is shown, but these parameters do not correlate linearly with the extrusion force. Viscosity at zero shear has a linear relationship with printing stability, but is weaker than melt stress. This is due to the possible structure of the paste during deformation. Some grease-based pastes can change during the extrusion process, resulting in additional friction and a measurable extrusion force [47].

The factor linking rheological properties to 3D printing behavior was also investigated. It was found that flow strength is a good indicator of printing stability. It was also found that formulations based on agar and other similar pastes have linear extrusion properties, while fat-based products behave differently. As a result, a rational approach to the development of printed formulations and design was proposed, which can achieve results faster, without unnecessary experimentation and waste of materials.

Therefore, further research in automated food 3D printing should focus on optimizing material composition and printing parameters to achieve the best possible results in terms of accuracy, quality and shape retention. This opens up new perspectives in innovative food technologies and the creation of customized, specialized products [42,43,44,45,46,47].

## 5. Advantages and Drawbacks

Some problems arose during the experiments. In particular, the researchers faced the need to optimize printing conditions for different proportions of ingredients, and they also studied the viscosity of edible inks and their properties. The 3D printing process has been found to significantly affect the bioactive compounds and antioxidant capacity of the resulting products [36,37,38,39,40]:

- Testing two 3D printing programs, the first provided higher stability of flavonols, chlorophyll A, chlorophyll B and carotenoids.
- Variations in 3D printing parameters, such as printing speed, line thickness, flow rate and nozzle distance, can affect the stability of bioactive compounds.
- Low printing speeds can promote the rapid degradation of chemical structures, which affects bacteriological stability.
- Three-dimensional printing programs do not affect antioxidant capacity as measured by the DPPH method, but they do affect capacity as measured by the FRAP method.

These findings underscore the importance of choosing 3D printing parameters to preserve the stability and antioxidant properties of bioactive compounds [36,37,38,39,40].

When selecting plant raw materials for 3D printing, it is important to consider physicochemical parameters [40]:

- The average water activity for the products created using 3D printing technology was 0.93%. The type and proportion of starch media have a significant impact on the water activity values of 3D printed products, as opposed to the type of 3D printing program. Three-dimensional printed products with wheat starch and its lower proportion (4% vs. 6% and 8%) had higher water activity. Increasing the proportion of hydrocolloids leads to lower water activity values due to free water binding and increased volume.
- The average pH of the 3D printed products was 3.30, and only the type of starch carrier had a significant effect on this indicator.

This underscores the importance of considering the type and proportion of starch carriers when developing new functional products using 3DP technology to ensure their quality. During the experiment, problems arose in optimizing 3D printing parameters for cookie production, namely selecting the best printing conditions, such as speed and nozzle diameter, to achieve optimal material extrusion speed. Using the wrong parameters can result in difficulties in extruding functional cookie material. In addition, it was important to properly evaluate changes in the size and structure of the cookies before and after processing to be sure that the process went correctly and geometric parameters were met [36,37,38,39].

During the experiment, dry samples containing orange peels gained higher hardness as a result of freezing and drying. It was noted that samples with a higher content of apricot pulp and lower content of orange by-products had lower gradients and higher average forces, which prevented them from being printed by the nozzle. This means that these samples were more difficult to print due to their higher viscosity and stiffness. It was also noted that the addition of orange peel caused color changes in the gels visible to the naked eye. In addition, after printing, the rheological parameters of the control samples differed from the uncontrolled samples, indicating changes in the structure and stability of the printed food [36,37,38].

The distribution of different water states in a lemon juice gel was determined using nuclear magnetic resonance, which is related to the structure of the material and its rheological properties. During the experiment, there were problems with the tail effect resulting from the discontinuity of extrusion. This effect caused the nozzle to break away from the object during printing on the plate, which disrupted the continuity of the material feed and affected the printing quality. The addition of the pea isolating protein (PIP) helped improve the flow characteristics of the 3D printing process and reduce the tail effect, which helped optimize shape retention and prevent extrusion problems. It was also found that the excessive addition of IBG led to the aggregation of proteins in the matrix, which led to the incomplete reproduction of the structure and fracture of the 3D printed line [41,42].

During the experiments, there were problems with raw production related to the excessive water content of the edible ink. As a result, the overall integrity of the printed forms deteriorated and was less stable. Types with high water content have difficulty printing and can easily collapse when layers are added. This is a serious problem because excess water can compromise the overall integrity of printed boards. The study also conducted tests to determine the ink limit voltage, which is important for the formation of self-stable structures. The plasticity property refers to the product’s ability to withstand deformation under pressure, which is also an important aspect in printing and extrusion [40]. During the experiment, problems arose in measuring the rheological properties of the orange peel ink components using an oscillating rheometer, as it was necessary to account for the excess material on the outside of parallel plates to prevent edge effects. In addition, there was a need to create a homemade software script in Python to convert the resulting G-code into MuCAD V code for further use on the DIW printer. In addition, work was conducted to measure the flavonoid levels in the 3D printed samples using liquid chromatography–mass spectrometry (LC/MS), which also presented challenges for compound extraction and analysis [36,37,38].

Experiments conducted using 3D printing technology have allowed researchers to identify challenges in optimizing printing conditions and studying the impact of the process on the bioactive compounds in the resulting products. It turns out that the choice of printing parameters, such as speed and line thickness, can significantly affect the stability of bioactive compounds. A low printing speed causes the rapid degradation of chemical structures, which negatively affects the stability of bioactive compounds. The type and proportion of starch carriers were also found to have a significant effect on the water activity of 3D printed products. Products containing less wheat starch were characterized by higher water activity. Increasing the proportion of hydrocolloids resulted in a decrease in water activity. The type of starch carrier was also found to affect the pH of 3D printed products, with products containing wheat starch having a lower pH than those containing corn starch. The research also showed that printing parameters, such as speed and nozzle diameter, affect the material extrusion process and changes in product dimensions and structure. For example, using the wrong parameters can result in difficulties in extruding the material and distorting the product geometry. In addition, the addition of various ingredients, such as orange peel or the pea isolating protein, also affects the properties and quality of printed products. For example, the addition of orange peel causes gels to become discolored, while an excessive amount of the pea isolating protein can cause protein aggregation and disrupt product structure.

Thus, the experimental results highlight the importance of selecting optimal 3D printing parameters to maintain the stability and antioxidant properties of bioactive compounds. When selecting plant raw materials and additives for 3D printing, it is also important to consider physicochemical parameters to ensure the quality and beneficial properties of the products. Such research will help improve 3D printing technologies and develop new functional products.

## 6. Future Remarks

Based on the above information, we can identify the main issues related to 3D food printing based on food waste. These include the following technical, nutritional and sustainability aspects:

1. Technical limitations. Current 3D food printing technology faces challenges in terms of speed, cost and ability to process the various textures that are critical to creating a variety of food products.
2. Material consistency. It is very important to get the right consistency when printing. Waste variability can make it difficult to create standard 3D printer materials.
3. Nutritional value. Ensuring that printed food products retain their nutritional value after processing is a major challenge, especially when using waste products, which can vary in quality.
4. Food safety. There are concerns about the safety and hygiene of the materials used in 3D food printing, which must meet strict food safety regulations.
5. Consumer acceptance. Gaining consumer acceptance for waste-based food products can be a challenge due to perceptions of food quality and safety.
6. Economic viability. The economic viability of the process must be demonstrated to make it a viable option for widespread use.
7. Regulatory hurdles. Allowing 3D printed food products to be consumed, especially those derived from waste streams, may raise regulatory issues.
8. Sustainability. While 3D printing has the potential to reduce food waste, it should also be evaluated for its overall environmental impact, including energy consumption and the life cycle of the materials used.

These challenges require continuous research and development to optimize 3D food printing processes, streamline material processing and ensure that the end products are safe, nutritious and acceptable to consumers. In the future, some of the above issues related to 3D food printing from food waste can be solved through research and development, as follows:

- Researchers can experiment with different 3D printing methods, such as extrusion printing, to improve the quality, control and speed of food printing. Innovations such as the development of a 3D printing platform that replicates the microstructure of real food can be tested and improved in the lab.
- Extensive research into the rheological properties of food waste is needed to improve its suitability for printing. For example, a study of potato peel waste evaluated its suitability for 3D printing by extrusion.
- There is a need for small-scale experiments in laboratories that can help understand the process parameters critical to scaling 3D printing to the industrial level. This includes optimizing processes that affect the quality and complexity of 3D printed food products.
- Food safety and compliance can be ensured through laboratory testing and certification processes. This includes testing for contamination and verifying that printed food products meet sanitary standards.
- Ethical and cultural effects can be investigated through consumer research and sensory evaluation in a controlled environment to assess acceptability and address potential problems.

By tackling these challenges, researchers can develop best practices and create a framework to address the challenges of 3D printing food from waste at a larger scale.

## 7. Conclusions

The analysis of existing 3DFP technologies revealed significant advancements in controlling and customizing food attributes such as nutrients, shape, texture, color and taste. These capabilities enable the production of personalized nutrition solutions, catering to individual dietary needs and preferences. The continuous improvement in the quality of printed foods underscores the potential of 3DFP to revolutionize the food industry. Research into large-scale production methods highlighted the critical need for scalable and cost-effective 3DFP techniques. Addressing challenges such as high production costs, limited material availability and stringent sanitation standards is essential for the widespread adoption of 3DFP. The successful scaling of 3DFP technologies will significantly enhance their commercial viability and accessibility. The study identified key challenges in the implementation of 3DFP, including production costs, material availability and sanitation standards. These challenges impact the broader adoption of 3DFP technologies. Overcoming these obstacles requires targeted research and development efforts to improve efficiency, material innovation and compliance with health and safety standards.

The study emphasized the critical importance of continuous research and development to address existing limitations and unlock new applications of 3DFP. Sustained investment in research and development is crucial for driving innovation, overcoming current challenges and enhancing the technological capabilities of 3DFP. This ongoing effort will ensure the continued evolution and improvement of 3DFP technologies. The evaluation of 3DFP’s revolutionary potential highlighted its transformative impact on the food industry. Moreover, 3DFP can contribute to the creation of a sustainable, personalized and high-quality food production system. By enabling the production of customized and nutritious food products, 3DFP has the potential to meet diverse consumer needs and address global food challenges, ultimately revolutionizing how food is produced and consumed.

## Data Availability

No new data were created or analyzed in this study. Data sharing is not applicable to this article.

## Supplementary Materials

The following supporting information can be downloaded at: https://www.mdpi.com/article/10.3390/foods13142186/s1, Figure S1: Results obtained by bibliometric analysis and clustering.

## References

[1] Waghmare R., Suryawanshi D., Karadbhajne S. (2023). Designing 3D printable food based on fruit and vegetable products—Opportunities and challenges. J. Food Sci. Technol..

[2] Seoane-Viaño I., Januskaite P., Alvarez-Lorenzo C., Basit A.W., Goyanes A. (2021). Semi-solid extrusion 3D printing in drug delivery and biomedicine: Personalised solutions for healthcare challenges. J. Control. Release.

[3] Grira S., Khalifeh H.A., Alkhedher M., Ramadan M. (2023). 3D printing algae-based materials: Pathway toward 4D bioprinting. Bioprinting.

[4] United Nations Reducing Food Loss and Waste: Taking Action to Transform Food Systems. https://www.un.org/en/observances/end-food-waste-day.

[5] Rogers H., Mohit S. (2021). Emerging Sustainable Supply Chain Models for 3D Food Printing. Sustainability.

[6] Silva V., Silva A., Ribeiro J., Aires A., Carvalho R., Amaral J.S., Barros L., Igrejas G., Poeta P. (2023). Screening of Chemical Composition, Antimicrobial and Antioxidant Activities in Pomegranate, Quince, and Persimmon Leaf, Peel, and Seed: Valorization of Autumn Fruits By-Products for a One Health Perspective. Antibiotics.

[7] Escalante-Aburto A., Trujillo-de Santiago G.T., Álvarez M.M., Chuck-Hernández C. (2021). Advances and prospective applications of 3D food printing for health improvement and personalized nutrition. Compr. Rev. Food Sci. Food Saf..

[8] Blutinger J.D., Tsai A., Storvick E., Seymour G., Liu E., Samarelli N., Karthik S., Meijers Y., Lipson H. (2021). Precision cooking for printed foods via multiwavelength lasers. NPJ Sci. Food..

[9] Le-Bail A., Maniglia B.C., Le-Bail P. (2020). Recent advances and future perspective in additive manufacturing of foods based on 3D printing. Curr. Opin. Food Sci..

[10] United Nations Треть прoдуктoв питания в мире выбрасывается. В ООН запустили глoбальную кампанию пo бoрьбе с пoтерей прoдoвoльствия. https://news.un.org/ru/story/2019/10/1364302.

[11] UPPRINTING FOOD Transforms Food Waste into Edible 3D Printed Snacks. https://www.voxelmatters.com/upprinting-food-food-waste-edible-3d-printed-snacks/.

[12] Zhao L., Zhang M., Chitrakar B., Adhikari B. (2020). Recent advances in functional 3D printing of foods: A review of functions of ingredients and internal structures. Crit. Rev. Food Sci. Nutr..

[13] VOSviewer-Visualizing Scientific Landscapes. https://www.vosviewer.com/.

[14] Godoi F.C., Bhandari B.R., Prakash S., Zhang M. (2018). Fundamentals of 3D Food Printing and Applications.

[15] Harasym J. (2022). 3D Printers for Food Printing—Advantages and Drawbacks of Market Ready Technical Solutions. Nauk. Inżynierskie I Technol..

[16] Lisovska T., Harasym J. (2023). 3D Printing Progress in Gluten-Free Food—Clustering Analysis of Advantages and Obstacles. Appl. Sci..

[17] Lisovska T., Banaś K., Orkusz A., Harasym J. (2023). Hydrothermal Treatment via Microwave Radiation Improves Viscoelastic Properties of Native Gluten-Free Flours for Extrusion 3D Printing. Appl. Sci..

[18] Markovinović A.B., Putnik P., Bosiljkov T., Kostelac D., Frece J., Markov K., Žigolić A., Kaurinović J., Pavlić B., Duralija B. (2023). 3D printing of functional strawberry snacks: Food design, texture, antioxidant bioactive compounds, and microbial stability. Antioxidants.

[19] Severini C., Derossi A., Ricci I., Caporizzi R., Fiore A. (2018). Printing a blend of fruit and vegetables. New advances on critical variables and shelf life of 3D edible objects. J. Food Eng..

[20] Abedini A., Hosseini H., Shariatifar N., Molaee-aghaee E., Sadighara P. (2024). Studying the impact of 3D printing technology on safety indicators of plant-based burger. Food Chem. X.

[21] Derossi A. (2021). 3D Food Printing: Opportunities, principles, limitations, and new ways in food production. IUFoST Sci. Inf. Bull. (SIB).

[22] Wang J., Zhang S., Ouyang Y., Li R. (2019). Current developments of bacteriocins, screening methods and their application in aquaculture and aquatic products. Biocatal. Agric. Biotechnol..

[23] Kewuyemi Y.O., Kesa H., Adebo O.A. (2022). Trends in functional food development with three-dimensional (3D) food printing technology: Prospects for value-added traditionally processed food products. Crit. Rev. Food Sci. Nutr..

[24] Pulatsu E., Lin M. (2021). A review on customizing edible food materials into 3D printable inks: Approaches and strategies. Trends Food Sci. Technol..

[25] Chachlioutaki K., Karavasili C., Mavrokefalou E.-E., Gioumouxouzis C.I., Ritzoulis C., Fatouros D.G. (2022). Quality control evaluation of paediatric chocolate-based dosage forms: 3D printing vs mold-casting method. Int. J. Pharm..

[26] Mantihal S., Prakash S., Bhandari B. (2019). Textural modification of 3D printed dark chocolate by varying internal infill structure. Food Res. Int..

[27] Waseem M., Tahir A.U., Majeed Y. (2023). Printing the future of food: The physics perspective on 3D food printing. Food Phys..

[28] Liu Z., Zhang M. (2019). 3D food printing technologies and factors affecting printing precision. Fundamentals of 3D Food Printing and Applications.

[29] Liu J., Sun L., Xu W., Wang Q., Yu S., Sun J. (2019). Current advances and future perspectives of 3D printing natural-derived biopolymers. Carbohydr. Polym..

[30] Liu Z., Bhandari B., Zhang M. (2019). Incorporation of probiotics (*Bifidobacterium animalis* subsp. *Lactis*) into 3D printed mashed potatoes: Effects of variables on the viability. Food Res. Int..

[31] Lu Y., Rewa R., Nitin N. (2023). Image-based assessment and machine learning-enabled prediction of printability of polysaccharides-based food ink for 3D printing. Food Res. Int..

[32] Kim Y., Kim H.W., Park H.J. (2021). Effect of pea protein isolate incorporation on 3D printing performance and tailing effect of banana paste. LWT.

[33] Taneja A., Sharma R., Khetrapal S., Sharma A., Nagraik R., Venkidasamy B., Ghate M.N., Azizov S., Sharma S., Kumar D. (2023). Value Addition Employing Waste Bio-Materials in Environmental Remedies and Food Sector. Metabolites.

[34] Hasan M.M., Islam M.R., Haque A.R., Kabir R., Kushe K.J., Hasan S.M.K. (2024). Trends and challenges of fruit by-products utilization: Insights into safety, sensory, and benefits of the use for the development of innovative healthy food: A review. Bioresour. Bioprocess..

[35] Yang F., Zhang M., Bhandari B., Liu Y. (2018). Investigation on lemon juice gel as food material for 3D printing and optimization of printing parameters. LWT.

[36] Tan J.D., Lee C.P., Foo S.Y., Tan J.C.W., Tan S.S.Y., Ong E.S., Leo C.H., Hashimoto M. (2023). 3D printability and biochemical analysis of revalorized orange peel waste. Int. J. Bioprint.

[37] Leo C.H., Lee C.P., Foo S.Y., Tan J.C.W., Tan J.D., Ong E.S., Hashimoto M. (2022). 3D printed nutritious snacks from orange peel waste. Mater. Today Proc..

[38] Molina-Montero C., Vicente-Jurado D., Igual M., Martínez-Monzó J., García-Segovia P. (2023). Fiber Enrichment of 3D Printed Apricot Gel Snacks with Orange By-Products. Gels.

[39] Jagadiswaran B., Alagarasan V., Palanivelu P., Theagarajan R., Moses J.A., Anandharamakrishnan C. (2021). Valorization of food industry waste and by-products using 3D printing: A study on the development of value-added functional cookies. Future Foods.

[40] Markovinović A.B., Brdar D., Putnik P., Bosiljkov T., Durgo K., Turković A.H., Karačonji I.B., Jurica K., Pavlić B., Granato D. (2024). Strawberry tree fruits (*Arbutus unedo* L.). Bioactive composition, cellular antioxidant activity, and 3D printing of functional foods. Food Chem..

[41] Tan J.D., Lee C.P., Leo C.H., Hashimoto M. (2022). Enhancing three-dimensional (3D) printablity of durian husk inks. Mater. Today Proc..

[42] Liu W., Chen L., McClements D.J., Peng X., Jin Z. (2023). Recent trends of 3D printing based on starch-hydrocolloid in food, biomedicine and environment. Crit. Rev. Food Sci. Nutr..

[43] Ahmadzadeh S., Clary T., Rosales A., Ubeyitogullari A. (2023). Upcycling imperfect broccoli and carrots into healthy snacks using an innovative 3D food printing approach. Food Sci. Nutr..

[44] Zheng Z., Zhang M., Liu Z. (2021). Investigation on evaluating the printable height and dimensional stability of food extrusion-based 3D printed foods. J. Food Eng..

[45] Armstrong C.D., Yue L., Deng Y., Qi D.H. (2022). Enabling direct ink write edible 3D printing of food purees with cellulose nanocrystals. J. Food Eng..

[46] Muthurajan M., Veeramani A., Rahul T., Gupta R., Anukiruthika T., Moses J.A., Anandharamakrishnan C. (2021). Valorization of Food Industry Waste Streams Using 3D Food Printing: A Study on Noodles Prepared from Potato Peel Waste. Food Bioprocess Technol..

[47] Zhu S., Stieger M.A., van der Goot A.J., Schutyser M. (2019). Extrusion-based 3D printing of food pastes: Correlating rheological properties with printing behavior. Innov. Food Sci. Emerg. Technol..

[48] Umeda T., Kozu H., Kobayashi I. (2024). Analysis of Pumpkin Paste Printability for Screw-Based 3D Food Printer. Food Bioprocess Technol..

[49] Jeon E.Y., Chun Y.G., Kim B.K. (2024). Investigation of carrot/squid blends as edible inks for extrusion 3D printing: Effect of hydrocolloids incorporation. J. Food Eng..

[50] Pant A., Leam P.X.N., Chua C.K., Tan U.-X. (2022). Valorisation of vegetable food waste utilising three-dimensional food printing. Virtual Phys. Prototyp..

