# Supplementary material for: Valorization of Fruit and Vegetables Industry By-Streams for 3D Printing—A Review
Source: Foods. 2024 Jul 11;13(14):2186. doi: 10.3390/foods13142186 (PMC11275474; doi:10.3390/foods13142186)
Supplement: Supplementary file 1 [file foods-13-02186-s001.zip › foods-3035183-supplementary.pdf]

foods

MDPI

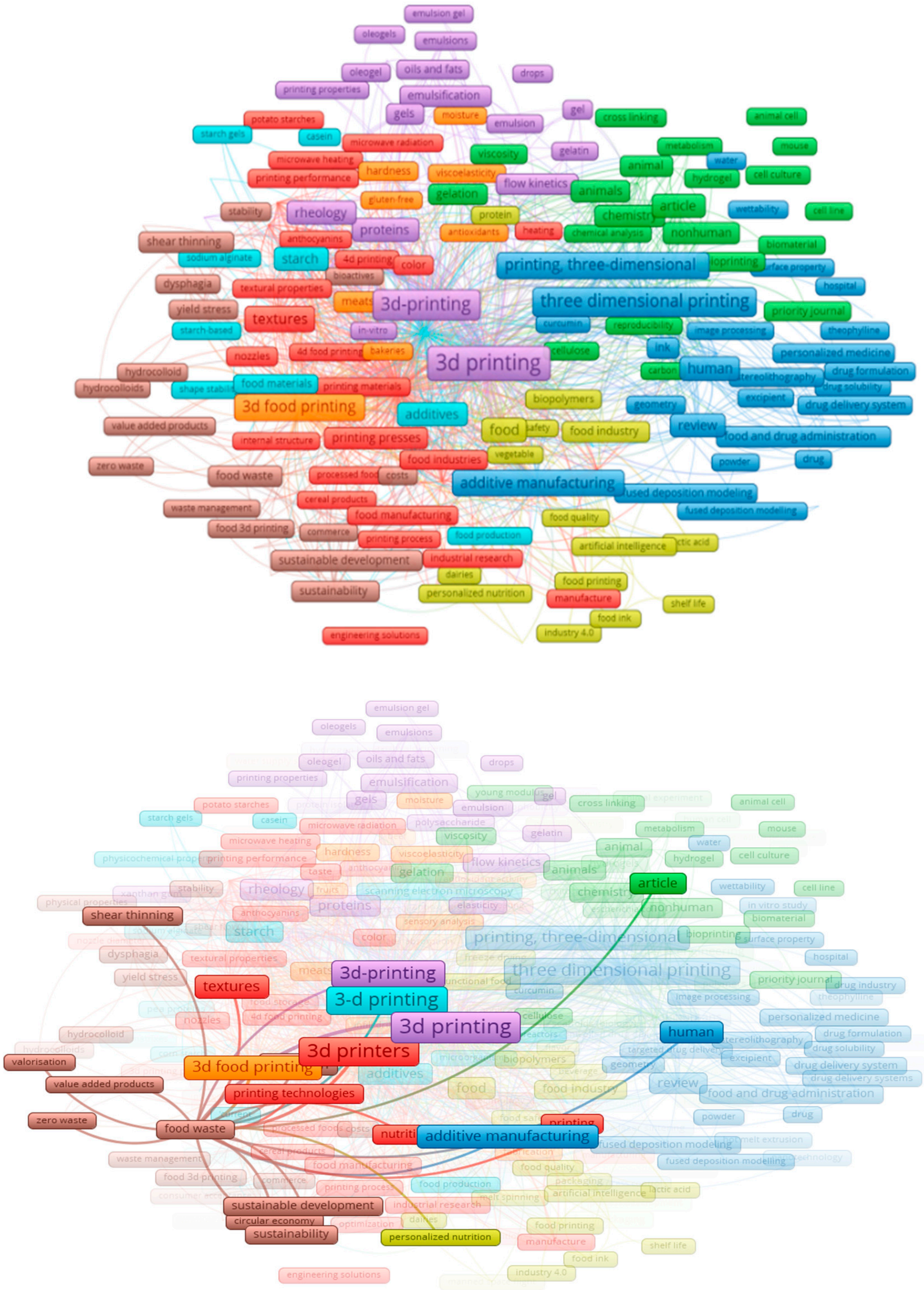

C1 - textures, printing presses, 4D printing, nozzle, color
C2 - biomaterial, animal, gelation, article, chemistry
C3 - three dimensional printing, additive manufacturing, human, review
C4 - food, food industry, biopolymers, vegetables, food ink
C5 - 3D printing, proteins, rheology, proteins, gel, emulsification
C6 - additives, food materials, starch, casein, food production
C7 - 3D food printing, meats, gluten-free, bakeries, hardness
C8 - food waste, value added products, food 3d printing, sustainable development.

Figure S1. Results obtained by bibliometric analysis and clustering.
